# Impact of Educational Intervention Concerning Awareness and Behaviors Relating to Avian Influenza (H5N1) in a High-Risk Population in Vietnam

**DOI:** 10.1371/journal.pone.0023711

**Published:** 2011-08-22

**Authors:** Toshie Manabe, Pham Thi Phuong Thuy, Vu Van Can, Jin Takasaki, Dinh Thi Thanh Huyen, Nguyen Thi My Chau, Takuro Shimbo, Bui Thi Thu Ha, Shinyu Izumi, Tran Thuy Hanh, Ngo Quy Chau, Koichiro Kudo

**Affiliations:** 1 Disease Control and Prevention Center, National Center for Global Health and Medicine, Tokyo, Japan; 2 NCGM-BMH Medical Collaboration Center, Hanoi, Vietnam; 3 Research Institute, National Center for Global Health and Medicine, Tokyo, Japan; 4 Bach Mai Hospital, Hanoi, Vietnam; 5 Health Department of Ninh Binh Province, Ninh Binh City, Vietnam; University of Liverpool, United Kingdom

## Abstract

**Background:**

Early initiation of treatment is essential for treatment of avian influenza A/H5N1 viral infection in humans, as the disease can lead to rapid development of severe pneumonia which can result in death. Contact with infected poultry is known to be a significant risk factor for contraction of H5N1 infection. However, handling and encountering poultry are a part of most peoples' daily lives, especially in rural communities in Vietnam where epidemic outbreaks among poultry have been continuously reported. Enhancing proper knowledge relating to H5N1 and to the importance of early initiation of treatment are crucial. The aim of this study was to develop an effective educational program to enhance awareness of H5N1 and motivate people to access to health care earlier when H5N1 infection is suspected or likely.

**Methodology and Principal Findings:**

A study was conducted in two agricultural communities (intervention and control groups) in the Ninh Binh province in Vietnam, where epidemic outbreaks of avian influenza have recently occurred in birds. A unique educational intervention was developed and provided to the intervention group, and no intervention was provided to the control group. A knowledge, attitude and practice (KAP) survey was conducted in both groups with a face-to-face interview by trained local healthcare workers at time points before and after the educational intervention. KAP scores were compared between the different time points and between the groups. How educational intervention influenced awareness relating to H5N1 and accessibility of healthcare in the population was analyzed. The study indicated an increased awareness of H5N1 and increased reliance on local health care workers.

**Conclusions:**

The novel educational program which was developed for this study impacted awareness of H5N1, and resulted in more people seeking early access to healthcare, and also resulted in earlier medical intervention for patients with H5N1 avian influenza infection in Vietnam.

## Introduction

Human infections with the highly pathogenic avian influenza A (H5N1) virus have been reported since November 2003 by countries in South-east Asia. The fatality rate is about 58% [1]. H5N1 infection can cause rapid development of severe pneumonia and can lead to acute respiratory distress syndrome [2]. Most cases of avian influenza in humans have resulted from contact with infected poultry, which is a significant H5N1 risk factor [3]. However, backyard poultry are common in rural communities in northern Vietnam, and epidemic outbreaks among poultry and non-domesticated birds have been continuously reported [4]. It is crucial for residents who live in high-risk areas to have a good understanding of why they should seek healthcare early once they experience symptoms.

The aim of the present study was to assess impact factors that can help raise awareness of H5N1 infection in order to develop an effective educational program to follow the strategy of treatment for H5N1 infection in humans, which includes early diagnoses and early medical intervention.

## Materials and Methods

### Study Sites

The study was performed in two communities: Yen Son and Ninh Hoa communes in the Ninh Binh province in Vietnam. The communes are physically separated and the populations attend geographically different venues and gathering. Ninh Binh province is located about 100 km south from Hanoi and has heavy traffic of people and goods due to a highway which passes through the province that connects north and south in Vietnam. Ninh Binh province has experienced epidemic outbreaks among birds and domesticated poultry[4]. A human mortality was reported there in 2008. The population and household numbers were 5,400 and 1,500 in the Yen Son commune and 5,600 and 1,700 in the Ninh Hoa commune, respectively. At the time of study the average income per capita in Yen Son and Ninh Hoa were $280 (US) and $240 (US), respectively.

### Participants

A total of 600 participants were randomly selected from four villages in the Yen Son commune (intervention group) and four villages in the Ninh Hoa commune (the control group). The sample size was chosen so that it could detect an effect of 0.25 with a power of 0.8 even after substantial dropout.

The participants lived in the study sites, and the age range of the participants was 18 to 80 years. Those in the intervention group participated in the educational campaign with their family and friends who lived in the Yen Son commune.

### Program of Educational Intervention

Educational intervention was provided four times to four groups in the intervention group and occured over 2 days at local meeting halls. The intervention was carried out by disseminating information through lectures, practical performances, educational songs and an interactive quiz game created by the study investigators. The lectures on general information about H5N1 infection were given by local healthcare leaders from the health department of Ninh Binh province. The educational intervention included distribution of leaflets and posters that conveyed educational messages to encourage people to access healthcare early, and development of a hygiene standard that included using masks, gloves and soaps. The local healthcare workers who worked at commune health centers received a training program from the study investigators before the educational interventions and surveys.

### Survey Methodology

The knowledge, attitude and practice (KAP) surveys were conducted in December 2009 as a pre-educational intervention survey (KAP 1) and 3 months after the educational intervention for the intervention group in March 2010 as a post-educational intervention survey (KAP 2). KAP 1 and KAP 2 for the control group were conducted at the same time point as the KAPs for the intervention group. The questionnaire was designed to assess knowledge, attitude and behaviors relating to H5N1 avian influenza infection, and was translated into Vietnamese. It collected information on demographic and socioeconomic measures, information sources for avian influenza, general knowledge of H5N1 infection, poultry handling and treatment seeking. The pilot survey was conducted at the Yen Thanh commune, Ninh Binh province in order to find applicable questions and interview methods for the community in Vietnam. All questions were either closed-ended or multiple choice. The questionnaire was collected during face-to-face interviews conducted by previously trained healthcare workers. The interview was held four times in single a day at the meeting halls in each of the four villages in order to avoid exchange of information about the contents of the interview among participants. For the evaluation of the educational intervention,a qualitative survey was done for randomly selected 16 subjects from study participants in the Yen Son commune after the educational intervention. The KAP scores were compared at the different time points and between the groups.

### Statistical Analysis

Data from the KAP surveys were double-entered by using Microsoft Access 2007 (Microsoft, Redmond, WA, USA) and analyzed by using STATA (StataCorp LP, College Station, TX, USA). The primary analysis was conducted to compare factors of the participants (e.g., age, gender, socioeconomic condition, education, source of information regarding avian influenza, relation to poultry) between the intervention and control groups using the Wilcoxon rank-sum test, chi-square test and Fisher's exact test. Economic conditions of subjects were divided into quintiles of family income and were qualified based on the possessions of assets such as a television, radio, telephone, water machine, refrigerator, buffalo, bicycle, motorbike, car and air conditioner.. Secondary analysis assessed differences of each categorical variable and frequencies between each group on KAP 1 and KAP 2 using the chi-square test. Knowledge and attitude-practice scores were calculated in accordance to their correct answers using the factor analysis adjusted to a mean of 50 and standard deviation of 10.

A multiple regression model was used to analyze possible influencing factors associated with KAP 1 and KAP 2 scores adjusted by initial score, age, gender, educational level, occupation and socioeconomic level. Subjects included in the calculations were participants who participated both on KAP 1 and KAP 2, and the calculations were adjusted by the KAP 1 score in order to eliminate the possibility of regression to the mean.

### Ethics

Study participants both in the intervention group and the control group provided written informed consent. Ethical approval was provided by the Institutional Review Board of the Ministry of Health, Vietnam, Bach Mai Hospital and the National Center for Global Health and Medicine, Japan.

We conducted the present study according to the Regulation on Organization and Operation of Ethical Committee in Biomedical Research, Ministry of Health-Vietnam and the Ethical Guidelines of Epidemiological Research by the Ministry of Health, Labor, and Welfare-Japan. We designed and performed our KAP survey with reference to “A GUIDE TO DEVELOPING KNOWLEDGE, ATTITUDE AND PRACTCE SURVERYS” by the World Health Organization.

## Results

### Educational intervention

Educational intervention was provided to the intervention group during the time between KAP 1 and KAP 2, and the total number of participants was 323. The data from those who participated in KAP 1 and the educational intervention were analyzed on KAP 2, so the number of participants decreased for the KAP 2.

Masks, gloves, and soap were used as the educational materials for the practical demonstration. Manuals for the educational campaign and presentation materials (including a DVD) for further campaigning which trained healthcare workers could conduct by themselves, were also developed. Educational intervention was evaluated by a qualitative survey administered by a face-to-face interview of 16 (6 of whom were female) participants in the Yen Son commune after the educational intervention for evaluation of the educational intervention. All subjects who took the qualitative survey were evaluated for their recollection of all of the contents of the educational intervention, and whether they reported the contents of the presentation to their relatives and friends.

### Knowledge, attitude, and practice (KAP) survey

The numbers of study participants who agreed to participate in the present study from four villages in the Yen Son commune (intervention group) and the Ninh Hoa commune (control group) were 417 (median age, 38 [IQR 26-50] y) and 418 (median age, 43 [IQR 32-57] y) in the pre-intervention survey (KAP 1), respectively and 264 (median age, 45 [IQR 18-79] y) and 288 (median age, 44 [IQR18-79] y) in the post-intervention survey (KAP 2), respectively. The socio-demographic characteristics of participants are shown in Table 1. The most common education level in the intervention and the control groups was primary/secondary school: 284 (68.5%) and 298 (71.3%) on KAP 1, 206 (71.8%) and 197 (76.7%) on KAP 2, respectively, and there was no significant differences between the groups. A difference was observed in occupation levels: 264 (63.7%) of participants were farmers in the intervention group vs. 330 (78.3%) in the control group on KAP 1 (P<0.001) and 177 (61.5%) in the intervention group vs. 212 (80.9%) in the control group were farmers on KAP 2 (P<0.001). A higher economic level (over average or above) was observed among the participants on KAP 2, and there was significance different between the groups (P<0.001 on KAP 1, P<0.001 on KAP 2).

**Table 1:** Background of study participants.

	KAP 1†			KAP 2‡		
	Yen Son	Ninh Hoa	P vaule*	Yen Son	Ninh Hoa	P value*
	(intervention)	(control)		(intervention)	(control)	
	(n = 417)	(n = 418)		(n = 288)	(n = 264)	
Characteristics	No. (%)	No. (%)		No. (%)	No. (%)	
**Gender - male (%)**	162 (38.9)	137 (32.8)	0.067	98 (34.0%)	66 (25.0%)	0.020^1)^
**Age – y**						
Median	38	43		45	44	
IQR	26–50	32–57		18–79	18–79	
**All patients - No./total No. (%)**			<0.001			0.003^2)^
<30	146 (35.0)	86 (20.6)		78 (27.1)	40 (15.2)	
30–49	152 (36.5)	183 (43.8)		108 (37.5)	117 (44.3)	
≥50	119 (28.5)	149 (35.7)		102 (35.4)	107 (40.5)	
**Education, No. (%)**			0.450			0.539^3)^
Illiterate	3 (1.2)	8 (1.9)		2 (0.7)	2 (0.8)	
Primary/Secondary school	284 (68.5)	298 (71.3)		206 (71.8)	197 (76.7)	
High school	114 (27.3)	100 (23.9)		64 (22.3)	49 (19.1)	
College/University	8 (2.0)	12 (2.9)		15 (5.2)	9 (3.5)	
**Occupation**			<0.001			<0.001^3)^
Farmer	264 (63.7)	330 (78.3)		177 (61.5)	212 (80.9)	
Employed worker	53 (13.0)	35 (8.3)		28 (9.7)	8 (3.1)	
Shop/Market	9 (2.2)	6 (4.3)		10 (3.5)	10 (3.8)	
Jobless	18 (2.2)	16 (3.8)		11 (3.8)	3 (1.2)	
Officer	14 (3.4)	18 (4.3)		8 (2.8)	18 (6.9)	
Others	59 (14.5)	17 (4.0)		54 (18.8)	11 (4.2)	
**Economic level** §			<0.001			<0.001^1)^
Lowest	59 (14.1)	109 (25.8)		37 (12.9)	37 (12.9)	
Low	66 (15.8)	108 (25.6)		35 (12.2)	35 (12.2)	
Average	92 (22.1)	51 (12.1)		117 (40.6)	117 (40.6)	
Over average	100 (24.0)	71 (16.8)		99 (34.4)	51 (19.3)	

* Frequencies between intervention and control groups were compared by ^1)^chi-square test, ^2)^wilcoxon rank-sum test, and ^3)^fisher's exact test.

† The knowledge, attitude and practice survey on before the educational intervention.

‡ The knowledge, attitude and practice survey on after the educational intervention.

§ Economic level was qualified based on the possessions of assets such as a television, radio, telephone, water machine, refrigerator, buffalo, bicycle, motorbike, car and air conditioner, and was divided into quintiles of family income.

**Figure 1:**
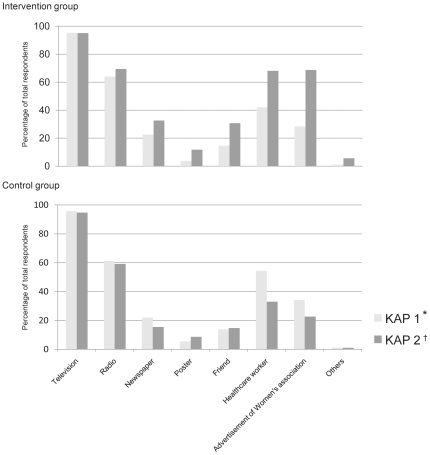
Information sources on avian influenza (H5N1). The changed percentage of information sources relating to avian influenza (H5N1) on *KAP 1 (pre-educational intervention survey) and †KAP 2 (post-educational intervention survey) at each group.

The information sources about avian influenza (H5N1) are listed in Table 2. The most common information source both for the intervention group and the control group was television: 95.9% and 95.7% of participants on KAP 1 (P = 0.869) and with 95.1% and 94.7% of participants on KAP 2 (P = 0.813), respectively, identified television as their main source of information. The source of information on avian influenza (H5N1) are shown in Figure 1. On KAP 1, 14.6% and 13.8% of participants in the intervention group reported receiving information from friends and healthcare workers, respectively (P = 0.756) and 42.0% and 54.3% of control group in the control group reported receiving information from friends and healthcare workers, respectively (P<0.001). On KAP 2, this changed changed in the intervention group to 30.6% and 14.8% (P<0.001) from friends and healthcare workers, respectively and in the control group 68.1% and 33.0% (P<0.001) from friends and healthcare workers, respectively.

**Table 2:** Sources of avian influenza information.

	KAP 1†			KAP 2‡		
	Yen Son	Ninh Hoa	P vaule*	Yen Son	Ninh Hoa	P value*
	(intervention)	(control)		(intervention)	(control)	
	(n = 417)	(n = 418)		(n = 288)	(n = 264)	
Information source§	No. (%)	No. (%)		No. (%)	No. (%)	
Television	400 (95.9)	400 (95.7)	0.869	274 (95.1)	250 (94.7)	0.813
Radio	266 (63.8)	256 (61.2)	0.448	200 (69.4)	156 (59.0)	0.011
Newspaper	94 (22.5)	92 (22.0)	0.853	94 (32.6)	41 (15.5)	<0.001
Poster	15 (3.6)	23 (5.5)	0.187	34 (11.8)	23 (8.7)	0.233
Friend	61 (14.6)	58 (13.8)	0.756	88 (30.6)	39 (14.8)	<0.001
Healthcare worker	175 (42.0)	227 (54.3)	<0.001	196 (68.1)	87 (33.0)	<0.001
Advertisement of women's association	118 (28.3)	143 (34.2)	0.065	198 (68.8)	60 (22.7)	<0.001
Others	4 (1.0)	5 (1.2)	0.740	16 (5.6)	3 (1.1)	0.004

* Frequencies between intervention and control groups were compared by chi-square test.

† The knowledge, attitude and practice survey on before the educational intervention.

‡ The knowledge, attitude and practice survey on after the educational intervention.

§ A multiple choices question, subjects can select more than one option.

Some of the information collected on the KAPs from the study participants is shown in Table 3. Most study participants from both groups had heard about avian influenza before the educational intervention (intervention group 98.6%, control group 98.3%, P = 0.797). Between the intervention and the control groups, listing H5N1 infection, malaria and tuberculosis listed as the most serious illness were 86.6% vs. 81.5%, (P = 0.046), 2.4% vs. 3.3%, (P = 0.424), and 3.1% vs. 4.7%, (P = 0.227) respectively on KAP 1, and 83.7% vs. 77.9%, (P = 0.095), 2.2% vs. 15.0%, (P<0.001), and 2.5% vs. 12.1% (P<0.001), respectively, on KAP 2. Over 80% of participants both on KAP 1 and KAP 2 knew that H5N1 transmitted from birds. The percentage of participants who said that they cook sick/dead chicken for eating in the intervention group was decreased from 2.2% to 1.8% after educational intervention. However, the percentage of participants who said that eat sick poultry was increased from 3.8% to 4.3%. On KAP 1, the percentage of participants who said that they washed hands after slaughtering poultry both in the intervention and control groups were 97.8%, vs. 96.0% (P = 0.118), respectively, and on KAP 2, this changed to 94.2% vs. 80.8% (P<0.001), respectively. The percentages of participants who said they would seek early access to healthcare after developing a fever and having contact with poultry in the intervention and control groups were significantly different on the post-intervention survey (P<0.001).

**Table 3:** Knowledge, attitude, and practice relating to avian influenza among study participants.

	KAP 1†			KAP 2‡		
	Yen Son	Ninh Hoa	P value*	Yen Son	Ninh Hoa	P value*
	(intervention)	(control)		(intervention)	(control)	
	(n = 417)	(n = 418)		(n = 288)	(n = 264)	
Valuable	No. (%)	No. (%)		No. (%)	No. (%)	
**Had heard of H5N1**	411 (98.6)	410 (98.3)	0.797	264 (100)	286 (99.2)	0.129
**Fright of illness (the most serious illness/symptom)**						
** Diarrhea**	32 (7.7)	45 (10.7)	0.134	28 (10.5)	58 (20.0)	0.003
** Cough/pneumonia**	12 (2.9)	24 (5.7)	0.045	5 (1.8)	38 (13.3)	<0.001
** Avian influenza (H5N1)**	361 (86.6)	341 (81.5)	0.046	221 (83.7)	224 (77.9)	0.095
** Malaria**	19 (2.4)	14 (3.3)	0.424	6 (2.2)	43 (15.0)	<0.001
** Tuberculosis**	13 (3.1)	20 (4.7)	0.227	7 (2.5)	35 (12.1)	<0.001
**Knowledge, attitude, and practice**						
**H5N1 transmit from birds**	369 (88.5)	369 (88.2)	0.879	213 (80.8)	268 (93.1)	<0.001
**Bury all dead poultry**	367 (88.0)	316 (75.6)	0.000	242 (91.7)	215 (74.6)	<0.001
**When you bury dead poultry, use protective clothes**	345 (82.7)	324 (77.5)	0.057	192 (72.8)	79 (27.5)	<0.001
**Cook dead chicken for eating**	9 (2.2)	10 (2.4)	0.837	5 (1.8)	6 (2.1)	0.823
**If your poultry die, report to authorities**	380 (91.1)	365 (87.4)	0.084	240 (90.9)	239 (82.9)	0.006
**If you find dead poultry, do not bring home**	415 (99.5)	409 (97.9)	0.035	264 (100)	285 (98.8)	0.062
**If you find dead poultry, touch them**	74 (17.7)	56 (13.5)	0.091	42 (15.9)	59 (20.4)	0.187
**Eat sick poultry**	16 (3.8)	21 (5.2)	0.338	11 (4.3)	16 (5.4)	0.573
**If you find dead poultry, throw them**	17 (4.1)	42 (10.0)	0.001	5 (1.8)	47 (16.3)	<0.001
**Wash hands after slaughter poultry**	408 (97.8)	401 (96.0)	0.118	249 (94.2)	233 (80.8)	<0.001
**If your family member has fever, ask him about contact with poultry**	389 (93.3)	380 (90.8)	0.177	257 (97.5)	252 (87.5)	<0.001
**Access to healthcare**						
**If you have a fever after contact with poultry, immediately seek treatment**	360 (86.3)	343 (82.0)	0.085	238 (90.2)	197 (68.3)	<0.001
**Where do you seek treatment for your fever after touching sick or dead poultry**						
** Commune health center**	284 (68.1)	263 (62.8)	0.106	197 (74.6)	177 (61.3)	0.001
** District hospital**	122 (29.3)	91 (21.8)	0.013	57 (21.7)	67 (23.3)	0.655
** Provincial hospital**	6 (1.4)	51 (12.3)	0.000	8 (2.9)	25 (8.8)	0.004

* Frequencies between intervention and control groups were compared by chi-square test.

† The knowledge, attitude and practice survey on before the educational intervention.

‡ The knowledge, attitude and practice survey on after the educational intervention.

A comparison of knowledge and attitude-practice scores on KAP 1 and KAP 2 did not reveal a big difference between the groups (Table 4). Even though the knowledge score was not different on KAP 2 between the groups, the p value of the attitude-practice score between the groups decreased on KAP 2. In order to investigate possible influencing factors associated with scores on KAP 1 and KAP 2, a multiple regression analysis (adjusted for initial score, age, gender, education level, occupation, and socioeconomic level) suggested that the knowledge score did not appear to be influenced by any of the above mentioned factors. Factors that influenced the attitude-practice score are shown in Table 5. The initial score, education level, and the intervention were factors which significantly influenced the attitude-practice score. Having a higher education level was strongly correlated with the largest difference in the attitude-practice score. The score of the intervention group differed by 8.69 points from that of the control group.

**Table 4:** Scores of knowledge and attitude-practice relating to avian influenza.

		Number	Mean	Median	25% tile	75% tile	P value*
**KAP 1** †	**Knowledge score^§^**						0.008
	Yen Son (intervention group)	417	50.36	53.34	51.60	53.34	
	Ninh Hoa (control group)	418	49.64	53.34	50.52	53.34	
	**Attitude-Practice score^§^**						<0.001
	Yen Son (intervention group)	417	51.29	54.58	50.72	55.21	
	Ninh Hoa (control group)	418	48.70	50.96	46.37	55.21	
**KAP 2** ‡	**Knowledge score** §						0.004
	Yen Son (intervention group)	288	50.04	51.60	50.07	51.60	
	Ninh Hoa (control group)	264	49.95	51.60	50.07	51.60	
	**Attitude-Practice score** §						<0.001
	Yen Son (intervention group)	288	54.45	57.41	52.37	57.58	
	Ninh Hoa (control group)	264	45.15	48.47	39.26	52.39	

* Frequencies between intervention and control groups were compared by Rank-sum test.

† The knowledge, attitude and practice survey on before the educational intervention.

‡ The knowledge, attitude and practice survey on after the educational intervention.

§ Knowledge and attitude-practice scores were calculated in accordance to their correct answers using factor analysis adjusted to a mean of 50 and standard deviation of 10.

**Table 5:** Results of multivariate analysis of the attitude-practice score.

	coefficient	Standard Error	P value	95% confident interval
Baseline value	−0.86	0.05	0.000	−0.95 − −0.77
Education				
Junior high school	12.69	3.43	0.000	5.96–19.43
High school	13.23	3.49	0.000	6.38–20.08
College/University	17.26	4.15	0.000	9.10–25.43
Yen Son commune	8.69	0.72	0.000	7.26–10.11

Adjusted R-squared 0.49

## Discussion

H5N1 influenza virus infection can be prevented by avoiding contact with infected poultry and non-domesticated birds. Previous reports from Vietnam, Thailand, Indonesia, and Cambodia showed the relationship between H5N1 human cases and contact histories with sick and/or dead poultries [5]–[8]. However, for people in agricultural communities in northern Vietnam, contact with poultry is a part of daily home and work lives. The rate of backyard poultry per total households was 90.9% in the Yen Son commune and 83.2% in the Ninh Hoa commune, and it is therefore difficult to avoid contact with poultry. Avian influenza (H5N1) infection in humans can develop rapidly into severe pneumonia [2]. Early initiation of antiviral treatment is recommended by the World Health Organization [9]. The treatment strategy requires motivation to visit a hospital in the early stage of the illness once symptoms develop. Furthermore, knowledge and awareness are important for seeking early access to healthcare and subsequent treatment of patients with H5N1 infections. In the present study, over 98% of subjects had ever heard of H5N1 avian influenza in both the intervention group and the control group (Table 3). The study was conducted in two communities in the same province. Although some statistically difference were seen in some aspects between groups both in KAP 1 and KAP 2, the study populations had similar socio-biographic and economical backgrounds in rural agricultural communities (Table 1). This differed from a previous study which was conducted in rural and urban areas in China [10]. So that, knowledge score did not appear to be influenced from the initial score, age, gender, education level, occupation, and socioeconomic level by a multiple regression analysis; however, the initial score, education level, and the intervention were factors which significantly influenced the attitude-practice score (Table 5). Having a higher education level was strongly correlated with the largest difference in the attitude-practice score.

Television was found to be the most effective way to disseminate information on avian influenza, followed by the radio, in the both groups (Table 2). General media reports also appeared to be an effective way to raise awareness and knowledge about avian influenza.

Education contributes significantly to prevention of illness. The educational programs that have been conducted by the government in Vietnam and by international organizations, as well as community-based advertisements that have been provided in the past few years appear to have been effective in reaching rural populations. A high degree of awareness and understanding of H5N1 infection was shown by both groups, even in the pre-educational intervention survey (Table 4). In addition to the surveys, educational intervention was conducted by disseminating information through lectures, practical performances, educational songs and an interactive quiz game. The material focused on motivating people to get early access to healthcare when they experience flu-like symptoms and have had close contact with infected poultry, and on raising awareness of influenza. The material was met to combine learning with fun for residents of a rural community, in order to maintain attention and to be easily understood. Lectures with general information about H5N1 infection were given by healthcare leaders from the health department of the Ninh Binh province, as they were advocators who were trusted and respected by the residents in the communities. The educational intervention included distribution of leaflets and posters, provided educational messages to encourage people to obtain early access to healthcare, and encouraged people to develop hygiene standards that included use of masks, gloves and soap. Changing behaviors and customs is difficult, especially for residents in rural areas with a one-time educational intervention. The present study indicated that habits such as touching and eating dead/sick poultry were reported on both KAP 1 and KAP 2 (Table 3). The main impact of the educational intervention in the present study was to increase the peoples' trust in local healthcare providers: increased percentages of healthcare workers and friends were listed as information sources on KAP 2 (Figure 1). Moreover, interest in avian influenza increased, as shown by slight differences between KAP 1 and KAP 2. Those are crucial factors which will lead people access healthcare when they feel something is wrong with their health. This could lead to more early diagnosis and early medical intervention.

Despite limitations in the study methodology due to the smaller number of participants for KAP 2 and due to the possibility of confounding factors during the study period for both the intervention and the control groups, the present study indicated the importance of involvement of local healthcare workers and administrators in H5N1 education and outreach. The novel educational program developed for this study had an impact on the awareness of H5N1 infection. Further educational intervention needs to be continuously conducted by local people, and can utilize television and radio. Furthermore, it might be possible to eventually change behavior related to avian influenza (H5N1) infection in Vietnam.

## References

[1] World Health Organization (2010). http://www.who.int/csr/disease/avian_influenza/country/cases_table_2010_06_08/en/index.html.

[2] The Writing Committee of the World Health Organization (WHO) Consultation on Human Influenza A/H5. (2005). Avian Influenza A (H5N1) Infection in Humans.. N Engl J Med.

[3] Dinh PN, Long HT, Tien NT, Hien NT, Mai le TQ (2006). Risk factors for human infection with avian influenza A H5N1, Vietnam, 2004.. Emerg Infect Dis.

[4] World Organization for Animal Health (2010). OIE daily update on avian influenza situation in birds.. http://www.oie.int/wahis/reports/en_fup_0000010146_20110110_20110110_121831.pdf.

[5] Hien TT, Liem NT, Dung NT, San LT, Mai PP (2004). Avian Influenza A(H5N1) in 10 Patients in Vietnam.. N Engl J Med.

[6] Chotpitayasuondh T, Ungchusak K, Hanshaoworakul W, Chunsuthiwat S, Sawanpanyalert P (2005). Human Disease from Influenza A(H5N1), Thailand, 2004. Emrg Infect Dis..

[7] Kandun IN, Wibisono H, Sedyaningsih ER, Yusharmen, Hadisoedarsuno W (2006). Three Indonesian Clusters of H5N1 Virus Infection in 2005.. N Engl J Med.

[8] Vong S, Ly S, Mardy S, Holl D, Buchy P (2008). Environmental Contamination during Influenza A Virus (H5N1) Outbreaks, Cambodia, 2006. Emrg Infect Dis..

[9] World Health Organization (2007). Clinical Management of human infection with avian influenza A (H5N1) virus.. http://www.who.int/csr/disease/avian_influenza/guidelines/ClinicalManagement07.pdf.

[10] Xiang N, Shi Y, Wu Jiabing, Zhang S, Ye M (2010). Knowledge, attitudes and practices (KAP) relating to avian influenza in urban and rural areas of China. BMC Infectious Diseases 10:34.. http://www.biomedcentral.com/1471-2334/10/34.

